# Swine wastewater drives dissemination of high-risk *bla*_NDM_-positive *Escherichia coli* clones

**DOI:** 10.3389/fmicb.2026.1842719

**Published:** 2026-06-10

**Authors:** Ying Chu, Yubao Li, Shuancheng Bai

**Affiliations:** 1 College of Agriculture and Biology, Liaocheng University, Liaocheng, Shandong, China; 2 College of Pharmaceutical Sciences and Food Engineering, Liaocheng University, Liaocheng, Shandong, China; 3 College of Smart Agriculture, Yulin Normal University, Yulin, China; 4 Guangxi Zhuang Autonomous Region Engineering Research Center of Facility Agriculture, Yulin, China; 5 Guangxi Colleges and Universities Engineering Research Center of Facility Agriculture, Yulin, China

**Keywords:** *bla*_NDM_, *E. coli*, MDR, public health, swine wastewater

## Abstract

**Introduction:**

Swine wastewater can accelerate the spread of antibiotic-resistant bacteria among animals, the environment, and humans. Therefore, the molecular epidemiological characteristics of carbapenemase-producing *Escherichia coli* isolates from swine wastewater was investigated.

**Methods:**

Antimicrobial susceptibility testing was used to determine metallo-enzyme activity. Conjugation experiments were used to determine transmissibility of the novel carbapenemase-encoding gene. Whole-genome sequencing (WGS) with short reads and ERIC-PCR genotyping were performed to characterize the genomic features of the isolates. Single nucleotide polymorphisms (SNPs) was used to determine strain relatedness.

**Results:**

A total of 100 non-duplicate carbapenemase-producing (*bla*_NDM-1_, *bla*_NDM-5_, and *bla*_OXA-48_-like) *E. coli* isolates were recovered from 316 swine wastewater samples. Of which, two isolates that co-harbored *bla*_OXA-48_-like and *mcr-1*, as well as five isolates that co-harbored *bla*_NDM-5_ and *tet(X4)*. All isolates were multidrug-resistant (MDR), with a majority exhibiting resistance to meropenem, ciprofloxacin, imipenem and gentamicin. In addition, WGS analysis further revealed that *bla*_NDM_ co-existed with other antibiotic resistance genes, conferring resistance to twelve classes of antimicrobials. These isolates were assigned to 12 distinct sequence types (STs), among which ST10 and ST5299 were the most prevalent. Additionally, Phylogenomic analysis revealed that clonal spread of *bla*_NDM_-positive ST10 *E. coli* isolates at swine wastewater in multiple cities, including Binzhou, Dezhou, Heze and Weihai. Meanwhile, a notable divergence in single nucleotide polymorphisms (SNPs) was observed in isolates from this study and public database, which indicates that these *bla*_NDM_-positive *E. coli* isolates have high genetic diversity.

**Discussion:**

This study underscores the importance of surveillance for *bla*_NDM_-positive microbes in swine wastewater, and continuous monitoring in swine wastewater is essential for ensure human and environmental health.

## Introduction

The presence of carbapenem-producing *Enterobacteriaceae* (CPEs) in swine wastewater is a growing concern because of their role in environmental contamination and their potential dissemination into human healthcare settings. These organisms often carry genes associated with intra- and inter-cellular gene mobility, virulence, metal tolerance, and antimicrobial resistance (Bai et al., 2022). Such a gene pool may interact with and modify the gene pool of environmental bacteria, and vice versa (Liu et al., 2024). Microorganisms resulting from these interactions may pose a health threat to livestock and poultry and, ultimately, to humans. Accordingly, CPEs in swine wastewater require greater attention.

Swine wastewater contains high levels of antimicrobial residues and acts as an important reservoir and dissemination vehicle for antibiotic resistance determinants (Liu et al., 2022). Clinically administered antimicrobials are rarely fully metabolized in organisms, and their degradation products are eventually discharged into the environment, thereby exacerbating environmental pollution and posing substantial public health risks. Furthermore, the overuse of *β*-lactam antibiotics promotes the enrichment and proliferation of CPEs in livestock and poultry breeding environments. Although antimicrobial resistance in pig farm ecosystems has been widely reported nationwide, existing studies lack systematic molecular characterization and phylogenetic tracing of CPEs derived from swine wastewater in Shandong Province. The epidemiological profiles, dominant sequence types (STs), and transmission potential of plasmid-mediated carbapenem resistance genes among local pig farms remain poorly characterized, representing an important knowledge gap (Ma et al., 2024).

The most common resistance mechanism in CPEs is the expression of *β*-lactam-hydrolyzing enzymes, such as New Delhi metallo-β-lactamase (NDM) and *Klebsiella pneumoniae* carbapenemase (KPC), encoded by *bla*_NDM_ and *bla*_KPC_, respectively. Unlike *bla*_KPC_, which is mainly found in bacteria from human infections (Fábio et al., 2021), *bla*_NDM_ has been identified in both humans and animals, including the swine wastewater environment (Cen et al., 2021; Wang et al., 2021a; Zhang et al., 2021). *bla*_NDM_ genes are generally located on mobile genetic elements (MGEs), such as plasmids, integrons, and transposons (Dossouvi and Ametepe, 2024). In addition to *bla*_NDM_, these MGEs frequently carry resistance genes associated with aminoglycosides, quinolones, and extended-spectrum β-lactamases (ESBLs), including *CTX-M* genes (Beig et al., 2023; Lo et al., 2022; Ramsamy et al., 2022). Consequently, antibiotic resistance genes (ARGs) may persist in aquatic environments, promoting further dissemination between aquatic environments and aquatic animals.

Studies have indicated that the prevalence of CPEs gradually increases along the farm-to-slaughter-to-retail supply chain of chicken and pig production systems in Chengdu, Sichuan Province, China. The corresponding positive rates were 4.70% (22/468) and 2.00% (7/349) at the breeding stage, 7.60% (19/250) and 22.40% (22/250) at the slaughtering stage, and 65.56% (158/241) and 34.26% (76/321) at the retail stage, respectively (Fu et al., 2024). Clonal dissemination of CPEs was also observed between farmed ducks (80.4%, 127/158) and slaughtered duck meat (82.2%, 37/45) in Anhui Province, China, including among samples originating from different farms (Guo et al., 2024). Such strains have also been detected across multiple environmental samples in Anhui Province, China, including farmland (10.3%, 8/78), vegetable fields (7.3%, 3/41), and long-term vacant chicken farm environments (25.6%, 41/160), indicating their persistence in these habitats (He et al., 2023). In addition, recent reports of plasmid-mediated *bla*_NDM_ or *bla*_OXA-48_-like genes co-harboring *mcr* and/or the *tet(X)* among *Enterobacteriaceae* pose a serious threat to public health and require urgent surveillance.

This study focused on multidrug-resistant (MDR) *Escherichia coli* isolated from swine wastewater in Shandong Province, China. The main objectives of the study were to assess the prevalence and molecular epidemiological features of these isolates, detect the co-harboring of *bla*_NDM-5_ and *tet(X4)* as well as *bla*_OXA-48_-like and *mcr-1* gene clusters, and evaluate the possible role of swine wastewater as a reservoir and dissemination pathway for these resistance mechanisms.

## Materials and methods

### Isolate collection

A total of 316 swine wastewater samples were collected from 29 pig farms across different regions in Shandong Province, China, on 3 June 2024. At least one pig farm was sampled in each region, and threee to five samples were collected from manure collection pits in at least two pig houses per farm. However, due to biosecurity control measures at the pig farms (to prevent contamination of pig herds by exogenous pathogenic microorganisms, entry to pig farms was prohibited for non-essential personnel), sufficient samples could not be obtained from every region.

Briefly, 1 mL of the original swine wastewater sample was added to 9 mL of Luria–Bertani broth for pre-enrichment without antibiotic selection. Approximately 10 μL of the pre-enriched bacterial suspension was then streaked onto MacConkey agar plates supplemented with 2.0 mg/L of meropenem using an inoculating loop, followed by incubation at 37 °C for 18 h (Use of MacConkey agar supplemented with 2 mg/L of meropenem may introduce selection bias for carbapenem-resistant isolates; therefore, the observed prevalence may not reflect the actual prevalence in the original samples).

A red colony was selected from each plate for identification. Species identification of bacterial isolates was performed using MALDI-TOF MS Axima™ (Shimadzu-Biotech Corp., Kyoto, Japan) coupled with the SARAMIS MS-ID database (version 4.18) and 16S rRNA sequencing. For carbapenem-resistant isolates, five major carbapenemase genes (*bla*_KPC_, *bla*_NDM_, *bla*_IMP_, *bla*_OXA-48_-like, and *bla*_VIM_) were detected by polymerase chain reaction (PCR) using previously described primers (Nisa et al., 2024).

### Antimicrobial susceptibility testing

The minimum inhibitory concentrations (MICs) of 14 antibiotics (cefotaxime, ceftazidime, aztreonam, amikacin, gentamicin, ciprofloxacin, tetracycline, tigecycline, fosfomycin, sulfamethoxazole/trimethoprim, imipenem, meropenem, colistin, and florfenicol) for all recovery isolates were determined by agar dilution and interpreted according to the Clinical and Laboratory Standards Institute (CLSI) guidelines (CLSI, 2024). Susceptibility to colistin and tigecycline was determined using the broth microdilution method, and results for *Enterobacteriaceae* were interpreted according to the 2024 European Committee on Antimicrobial Susceptibility Testing (EUCAST) breakpoint criteria. *E. coli* ATCC 25922 served as the quality-control strain for susceptibility testing.

### Conjugation assay

To determine the transferability of the resistance genes, streptomycin-resistant *E. coli* strain C600 was used as the recipient, and conjugation assays were performed using the filter-mating method. Transconjugants were selected on MacConkey agar plates containing both 1.0 mg/L of meropenem and 2000 mg/L of streptomycin. Transconjugants were confirmed by PCR (Wang et al., 2021b).

### WGS and phylogenetic analysis

Clonal relatedness among all *Enterobacteriaceae* isolates was analyzed by ERIC-PCR, as described previously (Huang et al., 2021). Genomic fingerprinting of all *E. coli* isolates was performed using ERIC-PCR. Amplified products were separated by agarose gel electrophoresis, and banding patterns were visualized and captured using a gel imaging system. The ERIC-PCR fingerprint profiles were subsequently analyzed by cluster analysis to evaluate genetic relatedness among isolates. Isolates presenting clearly distinguishable banding patterns were defined as non-clonal strains. A similarity threshold of 90% was set for genotype classification; isolates with a similarity coefficient below 90% were considered genetically distinct. The ERIC-PCR gel electrophoresis images were analyzed using Image Lab 5.0. To avoid redundant analysis of clonal duplicates, only one representative isolate from each unique ERIC-PCR banding pattern was selected for subsequent whole-genome sequencing (WGS), multilocus sequence typing (MLST), antimicrobial resistance gene detection, and plasmid analysis.

Genomic DNA from all non-clonal *bla*_NDM_-positive *Enterobacteriaceae* isolates identified by ERIC-PCR was subjected to 250-bp paired-end WGS using the Illumina MiSeq platform. Sequencing was performed with an average depth of ≥50 × and genome coverage of ≥95%. Raw reads were quality filtered by removing low-quality bases and adapter sequences to obtain clean data for downstream genomic analysis (Hu et al., 2022). Paired-end Illumina reads were assembled using SPAdes v4.2.0 (Prjibelski et al., 2020). Antibiotic resistance genes (ARGs) were predicted using ResFinder 3.1 (80% coverage and 60% identity thresholds)^1^. MLST and plasmid incompatibility types were analyzed using MLST 2.0^2^ and PlasmidFinder 2.1^3^ (95% coverage and 60% identity thresholds). The genome sequences of our isolates were used as input data.

Representative reference plasmid sequences were screened and downloaded from the National Center for Biotechnology Information (NCBI) database. BLAST Ring Image Generator (BRIG) was used to perform comparative genomic analysis and generate plasmid comparison maps to investigate genetic similarity and gene distribution among target plasmids. Specifically, genome sequences of the collected isolates were used as input, whereas highly homologous reference plasmids were systematically screened and retrieved from the NCBI database. BRIG software was then used to compare plasmid sequences and construct plasmid maps for analysis of plasmid genetic relatedness and gene organization. Plasmid mobility was evaluated based on the generated plasmid profiles.

The fragmented contigs carrying *bla*_NDM_ (and *bla*_OXA-48_-like) from all strains were initially compared using EasyFig software. Contigs of the same type were removed, and the remaining distinct contigs, together with highly homologous complete plasmid sequences retrieved from the NCBI database, were subjected to final genetic-environment analysis of the target genes using EasyFig (Sullivan et al., 2011).

A total of 12,193 *E. coli* strains were downloaded from the NCBI Pathogen Detection database^4^ as of September 2024. These strains were preliminarily classified according to isolation source, sampling year, and sampling region. Based on the sample size of each region, 397 strains were randomly selected at a ratio of 1:30. For regions with smaller numbers of available strains, the selection ratio was adjusted to 1:8–10.

All assembled genomes were used for core-genome alignment to generate a phylogenetic tree using Parsnp software from the Harvest suite (Gaviria et al., 2022). In this pipeline, putative recombinant sites were excluded using PhiPack 2.1.11 with a 100-bp sliding window, 10-bp step size, and a significance threshold of *p*-value of < 0.05. Only loci satisfying unified quality-control criteria were retained as reliable core-genome SNPs. Filtering thresholds were as follows: mapping quality (MQ) ≥30, genotype quality (GQ) ≥20, missing rate ≤10%, minor allele count (MAC) >3, and linkage disequilibrium (LD) pruning at *r*^2^ < 0.2. Sample quality-control criteria included mapping rate ≥80%, sequencing depth ≥10×, and contamination rate ≤5%.

Recombination analysis was subsequently performed using Gubbins, followed by removal of recombinant regions and phylogenetic tree construction using core-genome sequences (Bruen et al., 2006). The final set of core-genome SNPs was submitted to FastTree version 2.1.11 for reconstruction of a maximum-likelihood phylogenetic tree using default parameters (Poon et al., 2010).

Variant call format (VCF) files generated by Parsnp were used to determine pairwise single-nucleotide variant distances among the core genomes of all strains. For whole-genome alignment and phylogenetic analysis, the complete genome of *E. coli* K-12 MG1655 was used as the reference genome. Phylogenetic lineages were defined using HierBAPS v6.0 (Cheng et al., 2013). Heat maps were generated using R version 3.3.2 (R Foundation for Statistical Computing), and phylogenetic trees were visualized using FigTree v1.4.2 and iTOL v4 (Letunic and Bork, 2019).

## Results

### Prevalence of carbapenemase-producing *E. coli* isolates

A total of 316 swine wastewater samples were collected from pig farms across multiple cities in Shandong Province, including 20 samples from 4 farms in Dongying, 36 from 8 farms in Heze, 14 from 3 farms in Weifang, 71 from 15 farms in Dezhou, 14 from 3 farms in Tai’an, 14 from 3 farms in Yantai, 10 from 2 farms in Binzhou, 30 from 6 farms in Weihai, 28 from 6 farms in Jinan, and 64 from 13 farms in Liaocheng. Following selective enrichment, a total of 100 CPE isolates were recovered from these 316 samples, with an overall isolation rate of 31.65%.

Two types of CPE were identified in this study, including 98 *bla*_NDM_-positive strains and 2 *bla*_OXA-48-like_-positive strains, and no other carbapenemase-encoding genes were detected among these carbapenem-resistant isolates. Among the two *bla*_NDM_ variants, *bla*_NDM-5_ was dominant (94/98, 95.92%), followed by *bla*_NDM-1_ (4/98, 4.08%).

**Figure 1:**
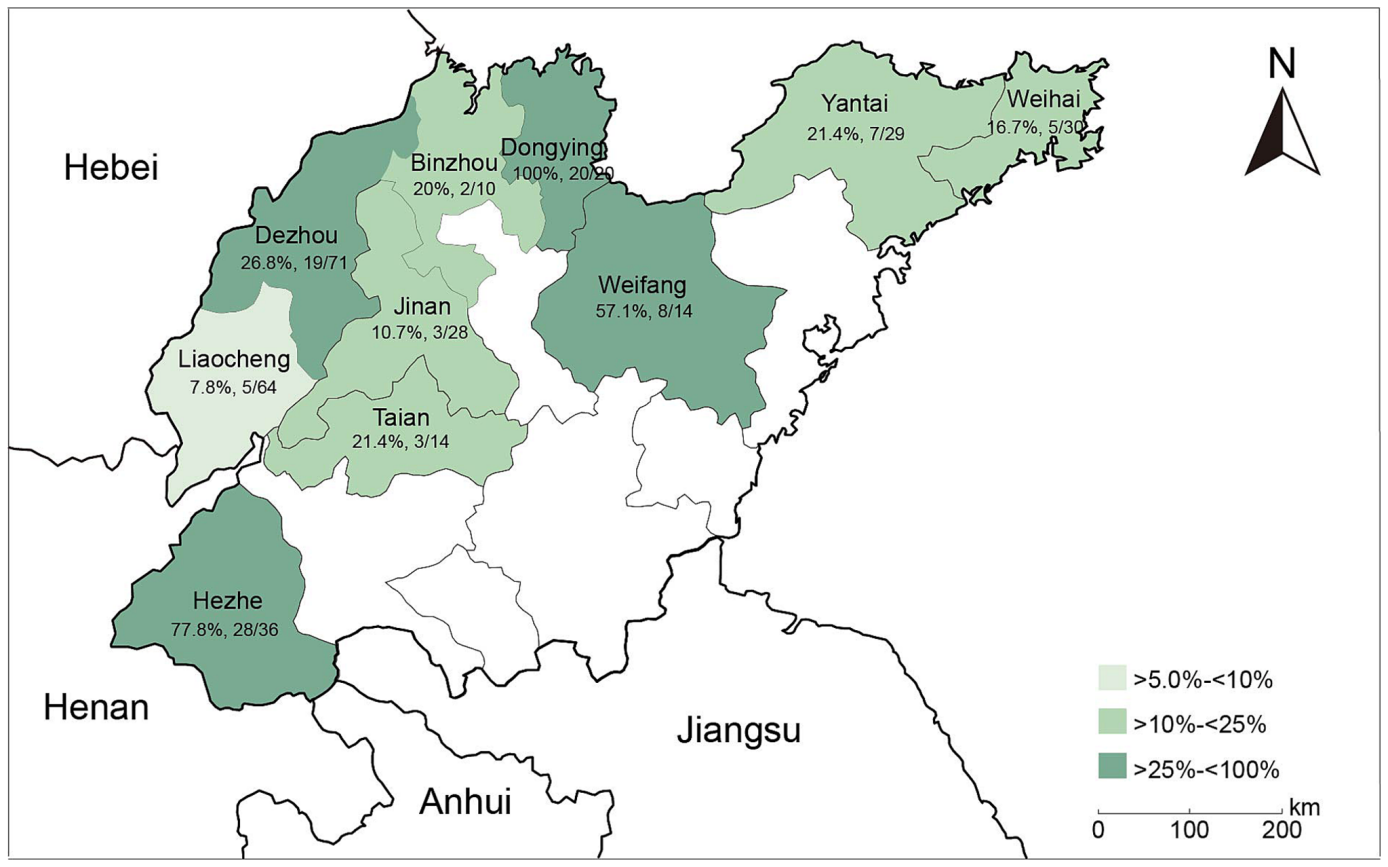
Geographical distribution of CPEs in Shandong Province, China. The prevalence of CPE varied significantly across different regions, ranging from 7.8% (Liaocheng) to 100% (Dongying), with a mean of 35.0% and a median of 21.4%. CPE, carbapenemase-producing *Enterobacteriaceae*.

The detection rate of CPE varied significantly among different cities in Shandong Province. Dongying exhibited the highest detection rate at 100% (20/20), followed by Heze (77.8%, 28/36), Weifang (57.1%, 8/14), and Dezhou (26.8%, 19/71). By comparison, markedly lower detection rates were recorded in Taian (21.4%, 3/14), Yantai (21.4%, 3/14), Binzhou (20.0%, 2/10), Weihai (16.7%, 5/30), Jinan (10.7%, 3/28), and Liaocheng (7.8%, 5/64), with Liaocheng showing the lowest prevalence across all surveyed regions (Figure 1).

### Antibiotic resistance phenotypes

**Figure 2:**
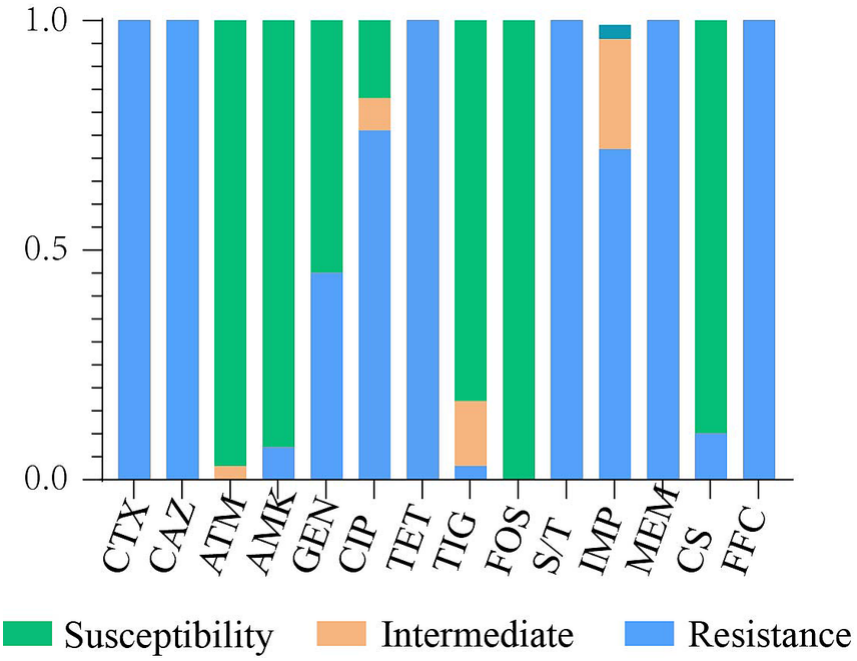
Minimum inhibitory concentrations of tested antimicrobial agents for the studied bacterial isolates. CTX, cefotaxime; CAZ, ceftazidime; ATM, aztreonam; AMK, amikacin; GEN, gentamicin; CIP, ciprofloxacin; TET, tetracycline; TIG, tigecycline; FOS, fosfomycin; S/T, sulfamethoxazole/trimethoprim; IMP, imipenem; MEM, meropenem; CS, colistin; FFC, florfenicol.

All 100 non-duplicate CPE isolates showed phenotypic resistance to meropenem, cefotaxime, ceftazidime, tetracycline, and trimethoprim-sulfamethoxazole. This uniform resistance profile is expected because the strains were recovered using selective culture protocols targeting carbapenem-resistant isolates (Figure 2; Supplementary Table S1).

In addition, the majority of these isolates were resistant to ciprofloxacin (76/100, 76%), imipenem (72/100, 72%), and gentamicin (45/100, 45%). In contrast, lower prevalence rates of resistance were observed for colistin (10/100, 10%), amikacin (7/100, 7%), and tigecycline (3/100, 3%). Notably, none of the isolates showed resistance to aztreonam or fosfomycin. Given that all strains were recovered through carbapenem-based selective screening, the low colistin resistance rate observed in this cohort is biologically plausible and not unexpected.

### Phylogenetic analysis of blaNDM and blaOXA-48-like-positive *E. coli* isolates

**Figure 3:**
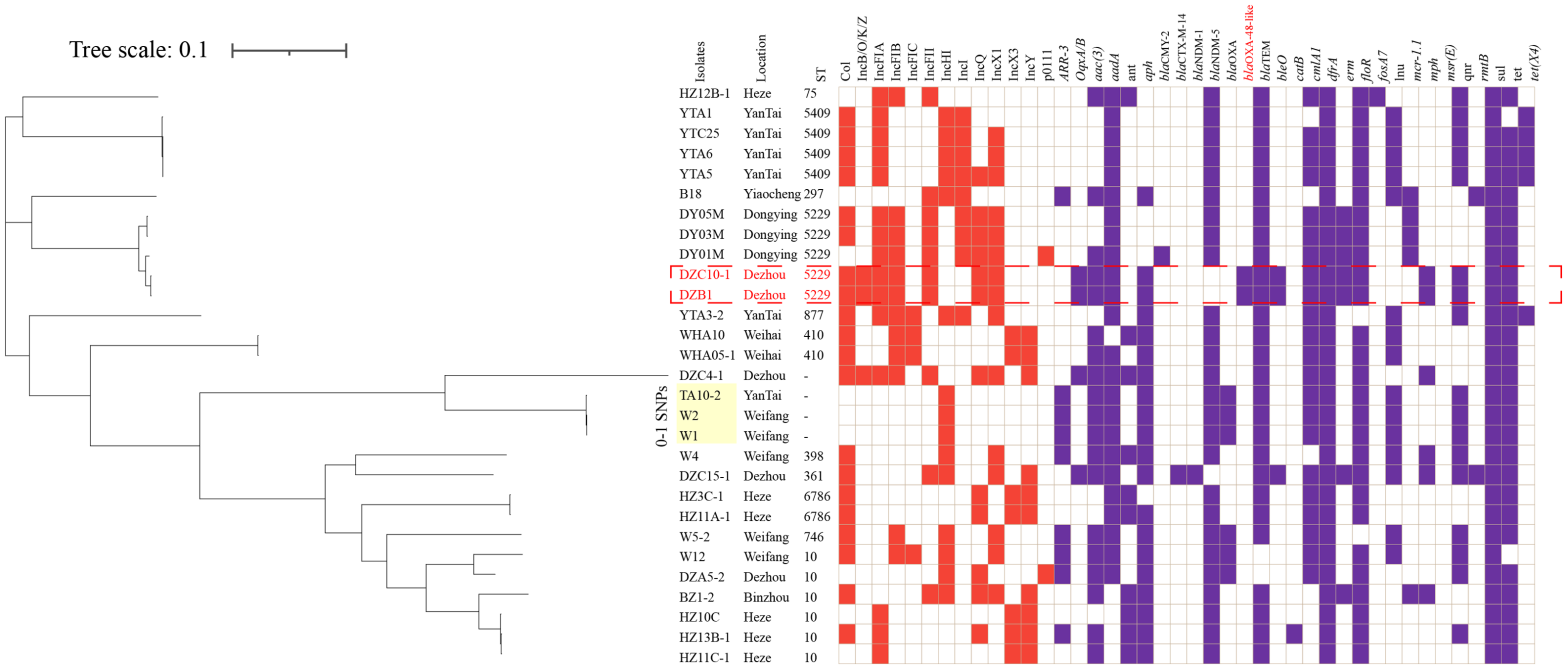
Phylogenetic analysis of CPE isolates in this study. A Bayesian evolutionary tree was constructed using core-genome SNPs. Each isolate is labeled with the city, year of isolation, and ST. The red-filled squares indicate the presence of the indicated ARGs. Blue indicates the presence of the target resistance gene (otherwise absent), whereas red indicates the presence of the incompatibility group (otherwise absent). CPE, carbapenemase-producing *Enterobacteriaceae*; SNPs, single nucleotide polymorphisms; ST, sequence type; ARGs, antibiotic resistance genes.

Based on preliminary ERIC-PCR genotyping, 29 *E. coli* isolates representing distinct genetic banding patterns, diverse geographic origins, and antimicrobial resistance profiles were selected for subsequent WGS. Results revealed that these isolates belonged to 12 distinct STs, whereas 4 isolates remained unclassified. Overall, ST10 (20.7%, 6/29) was the most prevalent and was identified in Binzhou, Dezhou, Heze, and Weihai, followed by ST5229 (17.2%, 5/29), which was identified in Dongying and Dezhou. These findings suggest geographic clustering of specific STs (Figure 3; Supplementary Table S2).

A phylogenetic tree constructed using these CPE isolates revealed that all *E. coli* isolates clustered into three distinct lineages. Lineage I mainly comprised ST5409 isolates originating from Taian, with these isolates sharing only 0–2 core-genome SNPs (cgSNPs). Lineage II mainly consisted of ST5229 isolates from Dongying and Dezhou. However, these isolates exhibited significant SNP differences (1,314 SNPs), indicating that they were not clonally related. Similarly, strains from Dezhou carried *bla*_OXA-48_-like gene differed by 36 cgSNPs.

In addition, lineage III mainly comprised ST10 isolates originating from Weifang, Bingzhou, Dezhou, and Heze. Three newly identified strains that could not be assigned to known STs differed by only one SNP from strains identified in Tai’an and Weifang.

Overall, phylogenomic analysis demonstrated substantial variation in the core genomes of *bla*_NDM_-positive *E. coli* isolates, indicating high genetic diversity among isolates recovered from swine wastewater in Shandong Province, China.

**Figure 4:**
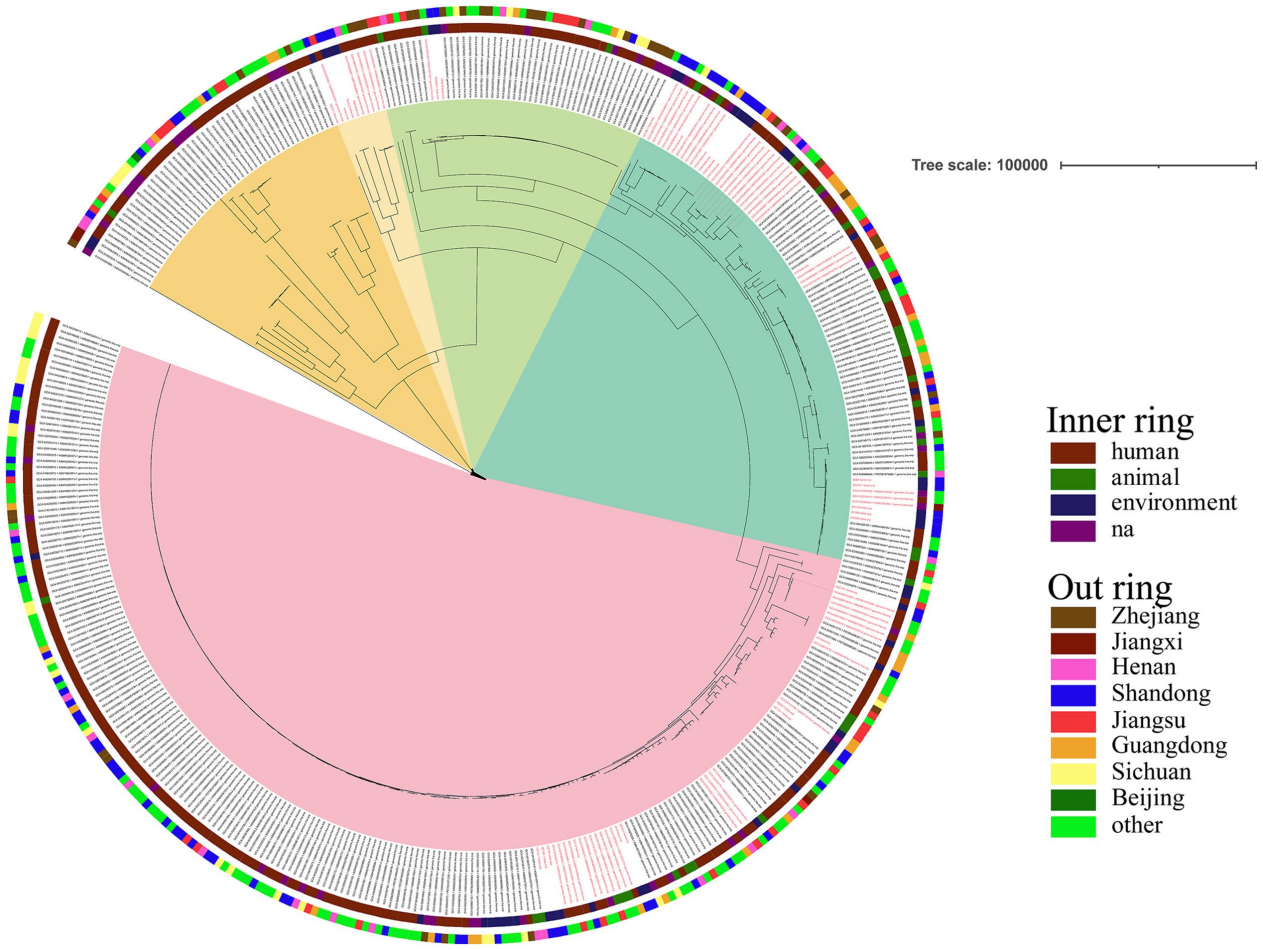
Phylogenetic structures of the *bla*NDM- and *bla*OXA-_48_-like-positive *Escherichia coli* isolates from this study and the GenBank database. Strain hosts and countries of origin are indicated in the inner and outer rings, respectively. NDM, New Delhi metallo-β-lactamase.

To further assess the relationship between isolates from the current study and publicly available isolates in China, a total of 397 *bla*_NDM_-positive *E. coli* isolates obtained from 32 provincial-level administrative regions were randomly selected from the National Center for Biotechnology Information (NCBI) database (as of April 2024) (Figure 4; Supplementary Table S3). Notably, NCBI-derived datasets inevitably contain sampling bias and uneven geographic representation; therefore, this comparison was only used for preliminary genetic reference analysis rather than unbiased population inference.

A maximum-likelihood phylogenetic tree was then constructed using these 426 *bla*_NDM_-positive *E. coli* isolates, which were grouped into 6 clades, among which the 29 isolates from this study were distributed across 5 clades. In addition, notable SNP divergence was observed between the *bla*_NDM_-positive *E. coli* isolates analyzed in this study and those obtained from the NCBI database, indicating high genetic diversity among these *bla*_NDM_-positive *E. coli* isolates.

### The genetic environments of blaNDM and blaOXA-48-like

**Figure 5:**
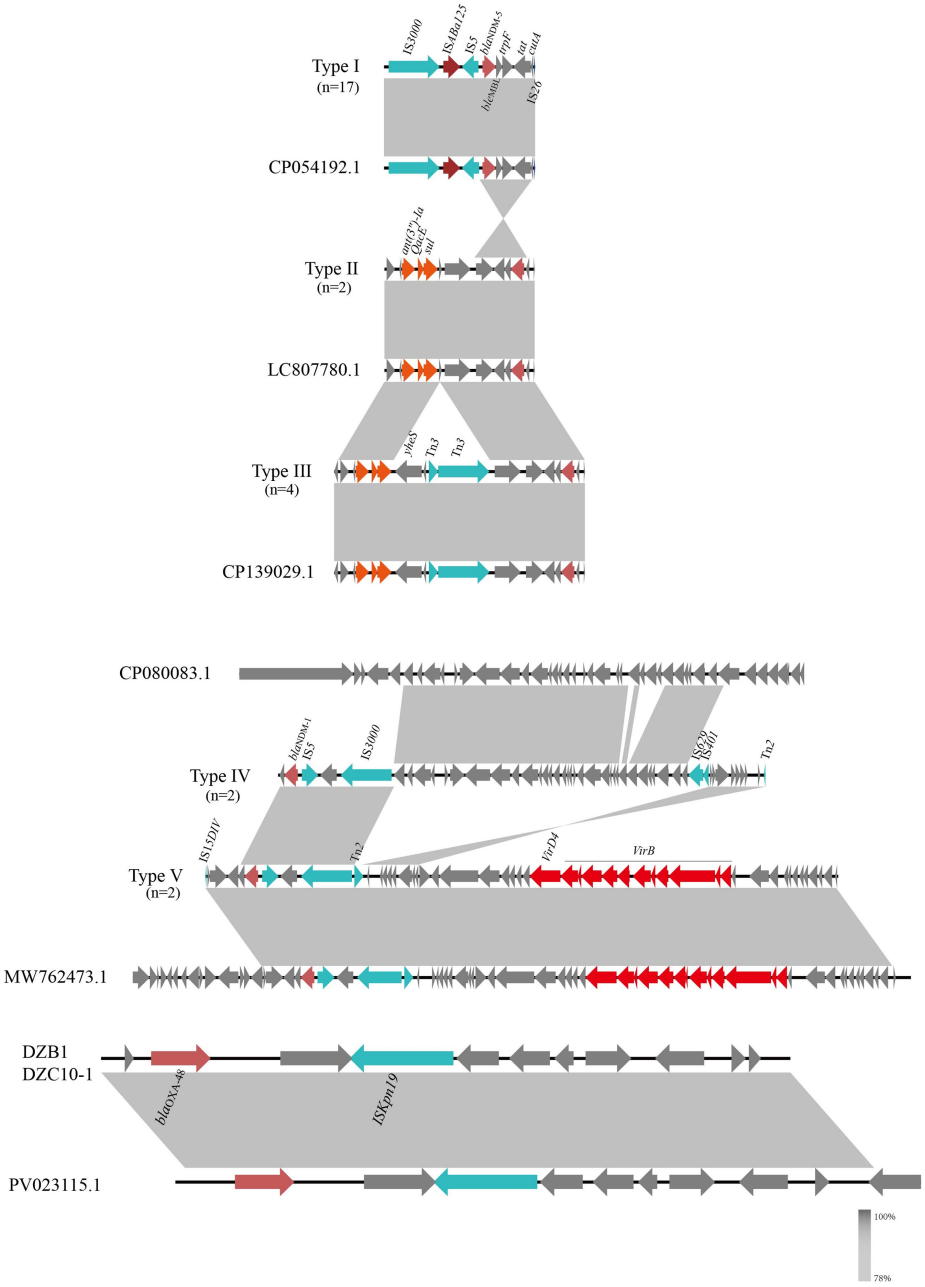
Genomic environments of *bla*_NDM_ and *bla*_OAX-48-like_ of *E. coli* isolates. The figure was generated using Easyfig. Regions of homology are marked by shading, and regions of ≥ 99.0% nucleotide sequence identity are shaded grey. Arrows indicate the direction of gene transcription. Red represents resistance gene, blue represents mobile genetic elements, black represents transfer gene *tra* clusters, and yellow represents other CDSs. CDSs, coding sequences.

A total of 5 genetic contexts (types I–V) were identified among the 27 CPE isolates. Classification was performed based on structural differences in the surrounding genetic environment of the *bla*_NDM_ and *bla*_OXA-48_-like gene within intact contigs (Figure 5).

Notably, *bla*_NDM-5_ was found in two out of the three genetic-context types. In all types, *bla*_NDM-5_ was directly associated with the *ble*_MBL_-*trpF*-*tat* genes. In addition, the Type I genomic context was the most prevalent *bla*_NDM_ genetic environment in this study (63%, 17/27), which was identical to a *bla*_NDM-5_-carrying plasmid (CP054192.1) identified in carbapenem-resistant *Enterobacteriaceae* isolates from humans in China (Yang et al., 2022). Meanwhile, the backbone structure containing IS*Aba125*-IS*5*-*bla*_NDM-5_-*ble*_MBL_-*trpF*-*tat*-IS*6* was usually carried by IncX3-type plasmids and was highly conserved (Lv et al., 2022).

In contrast, *bla*_NDM-1_ was linked to IS*5* and IS*3000* (Types IV and V). The Type IV genetic context resided within the backbone region of the IncY plasmid, and *bla*_NDM-1_ is flanked by IS*3000* and IS*5* within the backbone region of IncY plasmids (CP080083.1). This result was verified by PCR using primers designed within the backbone region of the IncY plasmid and regions adjacent to *bla*_NDM-1_. In Type V, the backbone carrying *bla*_NDM-1_ simultaneously harbored multiple virulence genes (*virB1-11* and *virD4*) and was identified in plasmid pHDC263-NDM (MW762473.1).

Types II and III were identical to pEC14-NDM-5 (LC807780.1) and shared a core resistance gene cluster (*ant(3′′)-Ia*-*qacE*-*sul*-*bla*_NDM-5_). However, Type III acquired unique evolutionary traits because of the insertion of the *yheS* gene (ATP-binding cassette transporter ATP-binding protein), mediated by the Tn*3* transposon. In addition, two *bla*_OXA-48_-like-positive bacterial strains identified in this study shared an identical genetic environment (*bla*_OXA-48_-like-IS*Kpn19*).

### Analysis of antibiotic resistance genes

We conducted a comprehensive antimicrobial analysis of all *E. coli* isolates, which revealed the presence of *β*-lactam resistance genes, including *bla*_NDM-1_ (3.45%, 1/29), *bla*_NDM-5_ (89.96%, 26/29), *bla*_OXA-10_ (20.69%,6/29), *bla*_OXA-48_-like (6.9%, 2/29), *bla*_CTX-M_ (3.45%, 1/29), *ble*_O_ (10.34%, 3/29), *bla*_CMY-2_ (3.45%, 1/29), and *bla*_TEM_ (93.10%, 27/29).

Other important resistance determinants conferring resistance to quinolones (*oqxA/B* [13.79%, 4/29] and *qnr* [58.62%, 17/29]), aminoglycosides (*rmtB* [6.9%, 2/29], *aadA* [82.76%, 24/29], *aph* [68.97%, 20/29], *ant* [34.48%, 10/29], and *aac* [65.52%, 19/29]), fosfomycin (*fosA7* [3.45%, 1/29]), chloramphenicol/florfenicol (*floR* [93.10%, 27/29] and *catB* [3.45%, 1/29]), sulfonamides (*sul* [100%, 29/29]), macrolides (*erm* [24.14%, 7/29] and *mph(A)* (20.69%, 6/29), rifampicin (*ARR-3* [31.03%, 9/29]), phenicols (*cmlA* [75.86%, 22/29]), lincomycin (*lnu* [51.72%, 15/29]), tetracycline (*tet* [93.10, 27/29]), and trimethoprim (*dfrA* [100%, 29/29]) were identified.

Additionally, we identified two isolates that co-harbored *bla*_OXA-48_-like and *mcr-1*, as well as five isolates that co-harbored *bla*_NDM-5_ and *tet(X4)* (Figure 3).

### Plasmid analysis

Using the PlasmidFinder 2.1 database for in silico analysis, a total of 13 incompatiblity-group plasmid replicon types were detected among the 29 carbapenemase-producing *E. coli* isolates, including Col (69%, 20/29), IncB/O/K/Z (10%, 3/29), IncFIA (52%, 15/29), IncFIB (41%, 12/29), IncFIC (14%, 4/29), IncFII (34%, 10/29), IncHI (52%, 15/29), IncI (31%, 9/29), IncQ (41%, 12/29), IncX1 (52%, 15/29), IncX3 (24%, 7/29), IncY (34%, 10/29), and p0111 (7%, 2/29).

It is worth noting that strains W1, W2, and TA10-2 from Weifang and Yantai carried only the IncHI plasmid, suggesting that this plasmid type represents an MDR plasmid harboring resistance determinants, including *bla*_NDM_. Subsequently, conjugation assays confirmed that the *bla*_NDM_ gene could be transferred into *E. coli* C600. PCR verification demonstrated that *bla*_NDM_ was exclusively located on the IncHI plasmid in transconjugant C600.

## Discussion

In the present study, we investigated the prevalence of carbapenemase-producing *E. coli* isolates from swine wastewater in Shandong, China. Our results revealed contamination of swine wastewater with *bla*_NDM_- and *bla*_OXA-48_-like-producing *Enterobacteriaceae* in the environment. Previous studies have also reported the detection of these species in the livestock-production environment (Wang et al., 2021b; Zhang et al., 2019). Studies have shown that Gram-negative bacteria, including *Enterobacteriaceae*, are able to survive on abiotic surfaces, which facilitates transmission from one surface to another, or even from the environment to patients and staff (Maaroufi et al., 2021), thereby potentially exacerbating the dissemination of *bla*_NDM_ and *bla*_OXA-48_-like.

Furthermore, all isolates in the present study displayed consistent resistance to the tested antibiotics. This phenomenon is not surprising, as our isolation protocol relied on selective enrichment and selective culture with carbapenem-containing media, which inherently screens for carbapenem-resistant strains. Consequently, the uniform resistance phenotype across all isolates is largely attributed to methodological selection bias rather than reflecting the natural resistance spectrum of *E. coli* in the general sample population. Future studies should adopt non-selective isolation approaches to better estimate the actual prevalence and resistance characteristics of circulating *E. coli* in the investigated region. More importantly, we detected *bla*_NDM_-positive *E. coli* with a relatively high prevalence in environmental samples (31%, 98/316), as well as two *bla*_OXA-48_-like-producing *E. coli* isolates. A high prevalence of *bla*_NDM_-positive and *bla*_OXA-48-_like positive has been reported in food animals, including chickens, cattle, and swine (Hayer et al., 2022; Liu et al., 2022). Together with the current findings, these data strongly indicate that the Chinese swine farm environment is likely to be an important reservoir for *bla*_NDM_ and *bla*_OXA-48_-like-carrying bacteria.

Importantly, carbapenems are not routinely applied in food-animal breeding. Previous studies have demonstrated that the high prevalence of CPE is closely associated with farm amoxicillin usage on farms (Ma et al., 2024). Accordingly, we speculate that the extensive application of amoxicillin on local farms likely imposed strong selective pressure, thereby facilitating the persistence and maintenance of CPE in this setting. In addition, swine wastewater is an open environment, which makes it difficult to perform complete disinfection and sterilization of the farm environment. This may contribute to widespread contamination by *bla*_NDM_-positive *Enterobacteriaceae* in farm environments. In this study, *bla*_NDM-5_ was the predominant variant, consistent with previous reports identifying *E. coli* as the primary *Enterobacteriaceae* species carrying *bla*_NDM-5_ in both farm animals and their environments (Cen et al., 2021; Wang et al., 2022).

In this study, the presence of *bla*_NDM_ and *bla*_OXA-48_-like genes was directly correlated with phenotypic resistance to carbapenem antibiotics among the tested isolates. Additionally, the correspondence between *mcr-1* detection and colistin resistance was assessed. Despite the identification of *mcr-1* in certain isolates, the phenotypic colistin resistance rate remained relatively low at only 10%. Furthermore, we evaluated the relationship between *tet(X4)* carriage and tigecycline susceptibility. Isolates carrying *tet(X4)* presented increased tigecycline MICs, confirming that the presence of *tet(X4)* was closely associated with reduced phenotypic susceptibility to tigecycline.

WGS analysis revealed that *bla*_NDM_ co-existed with 29 other types of ARGs, 12 of which were highly prevalent with detection rates >50%. This study revealed a high prevalence and diverse profiles of ARGs among the recovered *E. coli* isolates. In this study, *bla*_TEM_ and *bla*_NDM-5_ dominated the *β*-lactam resistance genes, with detection rates up to 93.10 and 89.66%, respectively, indicating the widespread dissemination of carbapenem and broad-spectrum β-lactam resistance determinants in the isolates. By comparison, other β-lactam resistance genes, including *bla*_NDM-1_, *bla*_OXA-10_, *bla*_OXA-48_-like, *bla*_CTX-M_, and *bla*_CMY-2_, were detected at relatively lower frequencies, suggesting their sporadic distribution in the tested strains. Notably, the isolates harbored abundant resistance genes covering multiple antibiotic categories. This diversity of ARGs confirms their role as potential sources of determinants of drug resistance (Liu et al., 2023). The sulfonamide resistance gene *sul* and the trimethoprim resistance gene *dfrA* were detected in all isolates (100%), implying an inherent and widespread MDR background in these *E. coli* isolates. High detection rates were also observed for *floR* (93.10%), *aadA* (82.76%), *cmlA* (75.86%), and *aph* (68.97%), which corresponded to the frequent application of phenicols, aminoglycosides, and other antimicrobial agents in livestock breeding. In contrast, several resistance genes, such as *rmtB*, *oqxA/B*, and *bla*_NDM-1_, presented low prevalence, reflecting limited local transmission of these rare resistance determinants (Wang et al., 2021b). Of particular concern was the co-occurrence pattern of critical resistance genes in individual isolates. We found that two isolates concurrently carried *bla*_OXA-48_-like and *mcr-1*, whereas five isolates co-harbored *bla*_NDM-5_ and *tet(X4)*. Such co-carriage of carbapenemase, colistin, and tetracycline resistance genes greatly facilitates the simultaneous spread of multiple resistance traits among bacterial populations, posing a severe threat to clinical antimicrobial therapy and public health.

To date, indeed, there are only limited reports of *bla*_OXA-48_ and *bla*_OXA-48_-like producing and *mcr-1* positive *E. coli* isolates in livestock (Aklilu et al., 2022; Carfora et al., 2022). In this study, WGS analysis revealed that *bla*_OXA-48_-like and *mcr-1* coexisted with 14 other types of ARGs. The widespread presence of carbapenem-resistant *Enterobacteriaceae* (CRE) and *mcr*-positive *E. coli* poses a huge threat to both animal and human health (Guo et al., 2023). In addition, *tet(X4)*-positive isolates have mostly been reported in animals such as pigs, chickens, cows and ducks, and *tet(X4)* has previously been identified in pigs and chickens at slaughters, as well as in soil and dust from animal farms and even pork from markets (Bai et al., 2019; Sun et al., 2019; Yu et al., 2021). However, although *tet(X4)* and *bla*_NDM-5_ genes have been reported to be co-harbored in *E coli* (Sun et al., 2021), it remains unknown whether *tet(X4)* and *bla*_NDM-5_ could co-exist in ST5409 and ST877. Here, we report the first identification of a carbapenem- and tigecycline-resistant *E. coli* isolate recovered from a pig farm in China harboring the *tet(X4)* and *bla*_NDM-5_ genes in different STs. This *E. coli* strain carries a large number of plasmids. The *tet(X4)*-positive *E. coli* isolates carried different replicon types, including Col, IncFIA, IncFIC, IncHI, IncI, and IncX1, and harbored multiple antimicrobial resistance genes, including *tet(X4)*, *bla*_NDM-5_, *aadA*, *bla*_TEM-1B_, *sul*, *floR*, and *qnrS1*.

## Conclusion

As far as we know, few studies have previously reported ST5299 *E. coli* co-harboring *bla*_OXA-48_-like and *mcr-1*, as well as ST5409 and ST877 *E. coli* isolates carrying the *bla*_NDM-5_-*tet(X4)* gene cluster. Although these isolates exhibited a relatively low prevalence in environmental samples, the carriage of transferable plasmid-borne resistance genes in these strains still represents a potential public health risk. Therefore, continuous monitoring of such MDR bacteria in humans, animals, and the environment should be considered to guide the implementation of public health interventions before clinical cases increase.

## Data Availability

The assembled genome sequences of the strains used in this study have been deposited in the GenBank database under BioProject number PRJNA1354101.

## Footnotes

1
https://cge.cbs.dtu.dk/services/ResFinder/

2
https://cge.cbs.dtu.dk/services/MLST/

3
https://cge.cbs.dtu.dk/services/PlasmidFinder/

4
https://www.ncbi.nlm.nih.gov/pathogens
