# Correction: Swine wastewater drives dissemination of high-risk *bla*_NDM_-positive *Escherichia coli* clones

**DOI:** 10.3389/fmicb.2026.1918252

**Published:** 2026-07-17

**Authors:** Ying Chu, Yubao Li, Shuancheng Bai

**Affiliations:** 1 College of Agriculture and Biology, Liaocheng University, Liaocheng, Shandong, China; 2 College of Pharmaceutical Sciences and Food Engineering, Liaocheng University, Liaocheng, Shandong, China; 3 College of Smart Agriculture, Yulin Normal University, Yulin, China; 4 Guangxi Zhuang Autonomous Region Engineering Research Center of Facility Agriculture, Yulin, China; 5 Guangxi Colleges and Universities Engineering Research Center of Facility Agriculture, Yulin, China

**Keywords:** *bla*_NDM_, *E. coli*, MDR, public health, swine wastewater

An incorrect number was provided for the Guangxi Natural Science Foundation. The correct number is “2026GXNSFHA00640358.”

The original version of this article has been updated.

