# Supplementary figures and images for: Swine wastewater drives dissemination of high-risk blaNDM-positive Escherichia coli clones
Source: Front Microbiol. 2026 Jun 10;17:1842719. doi: 10.3389/fmicb.2026.1842719 (PMC13290923; doi:10.3389/fmicb.2026.1842719)

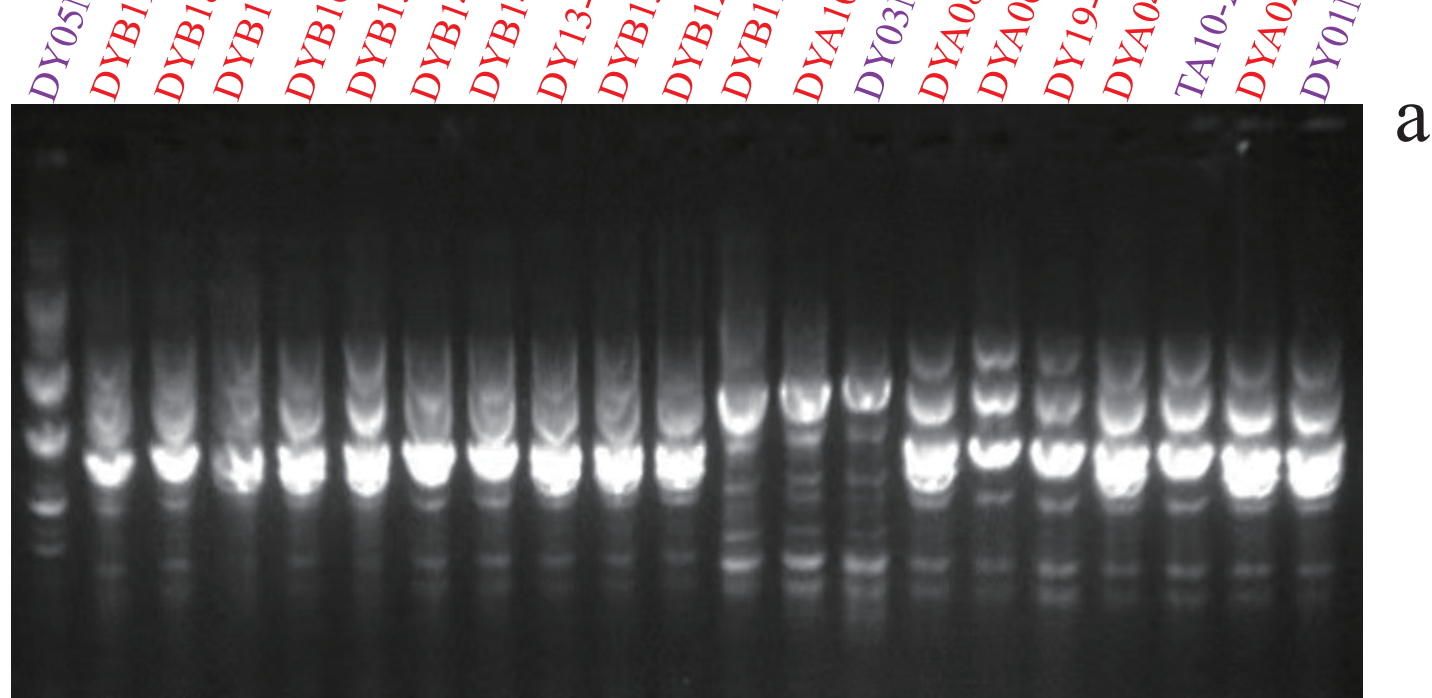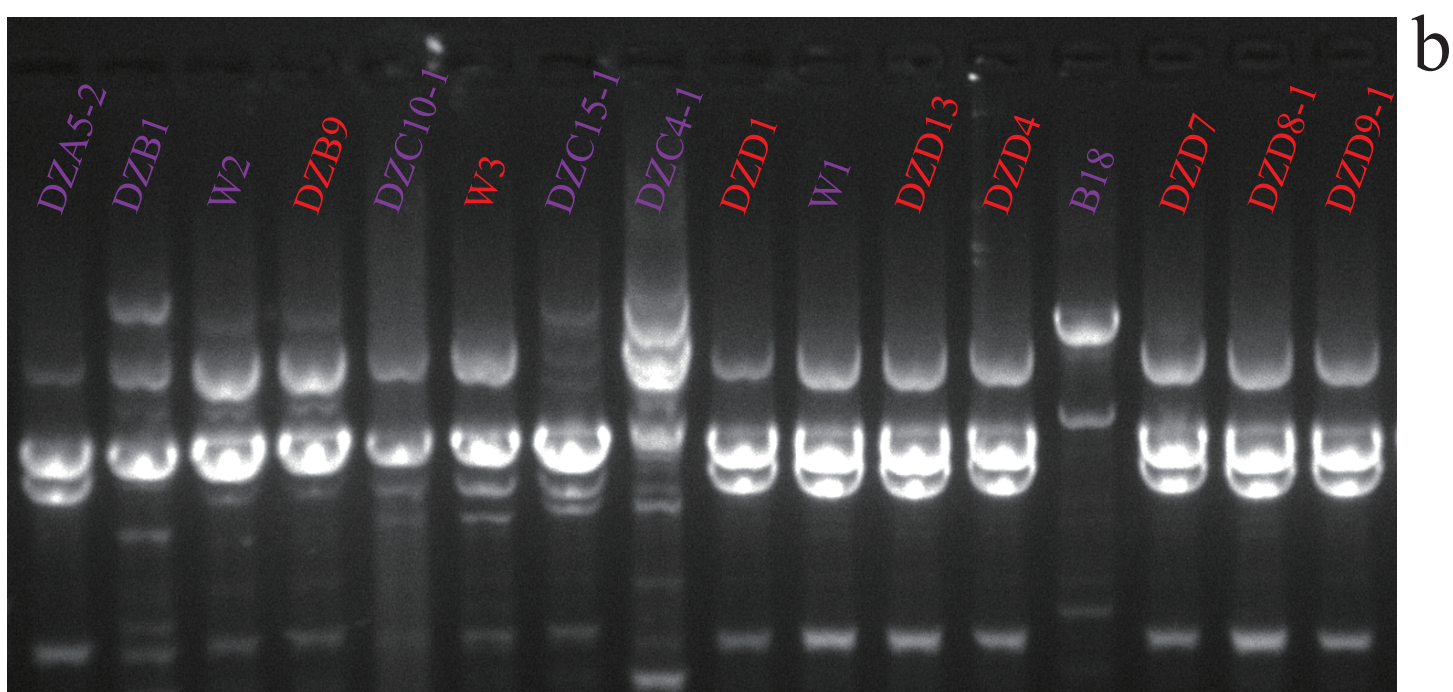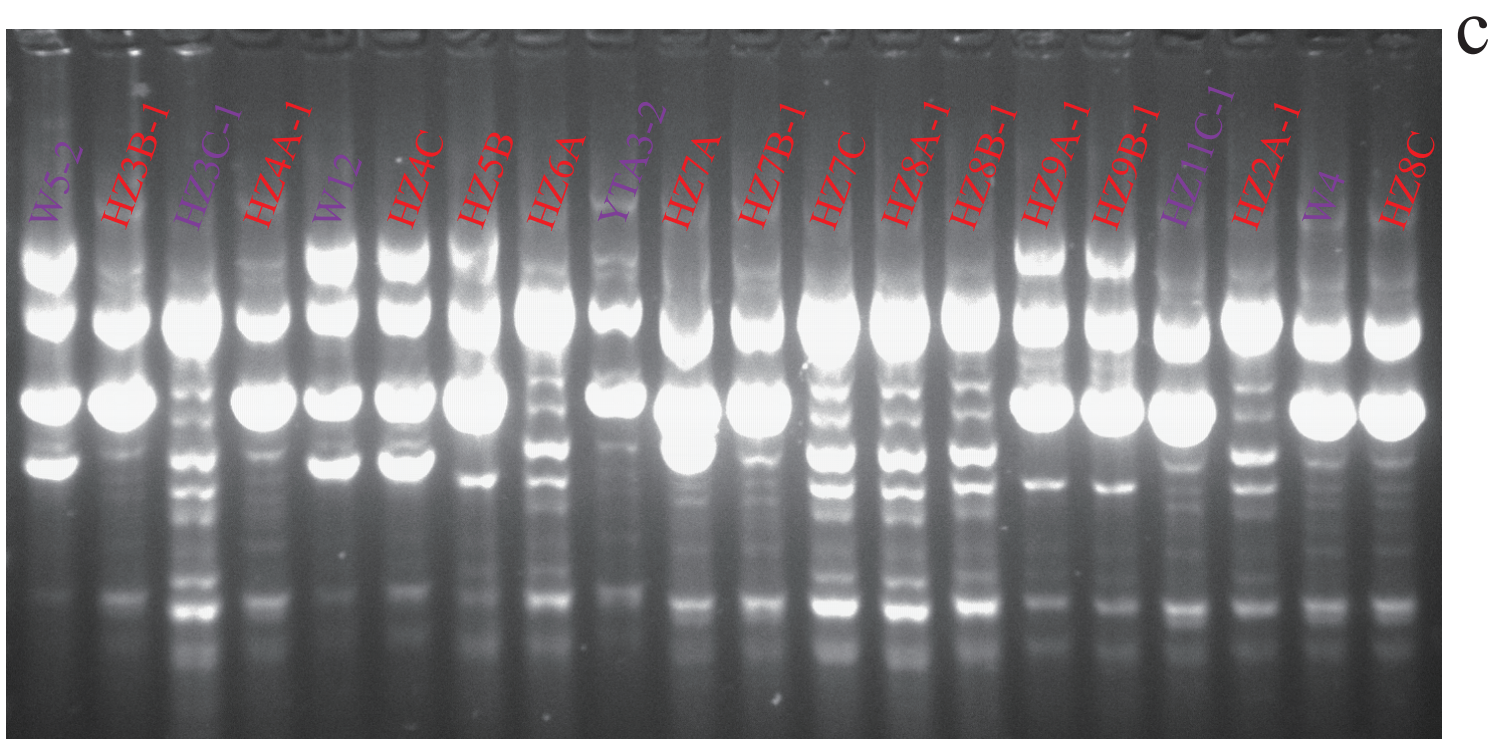

Supplement: Supplementary file 1 [file Data_Sheet_1.PDF]

d

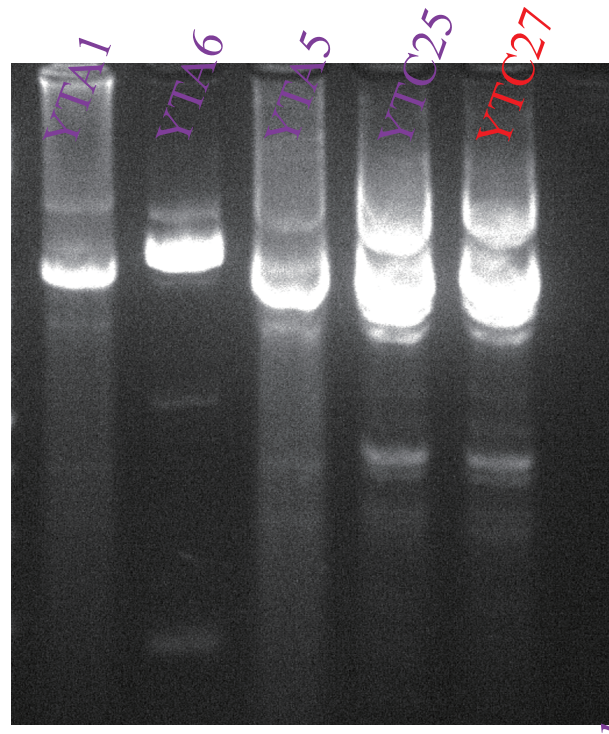

e

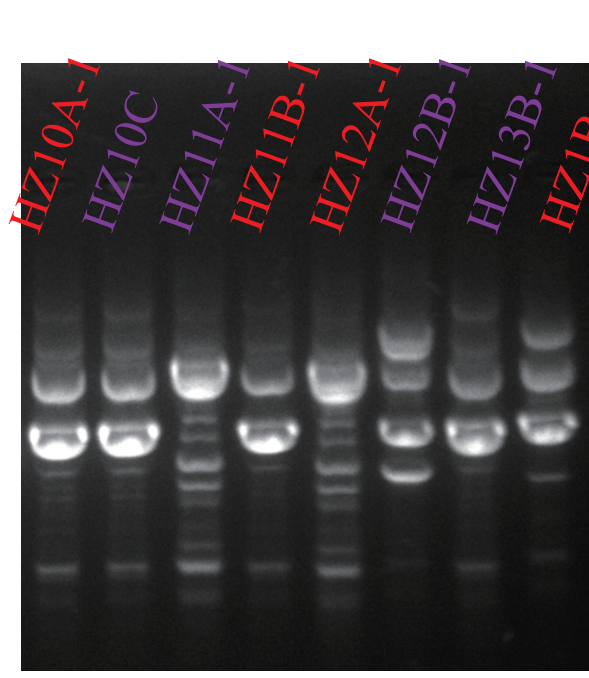

f

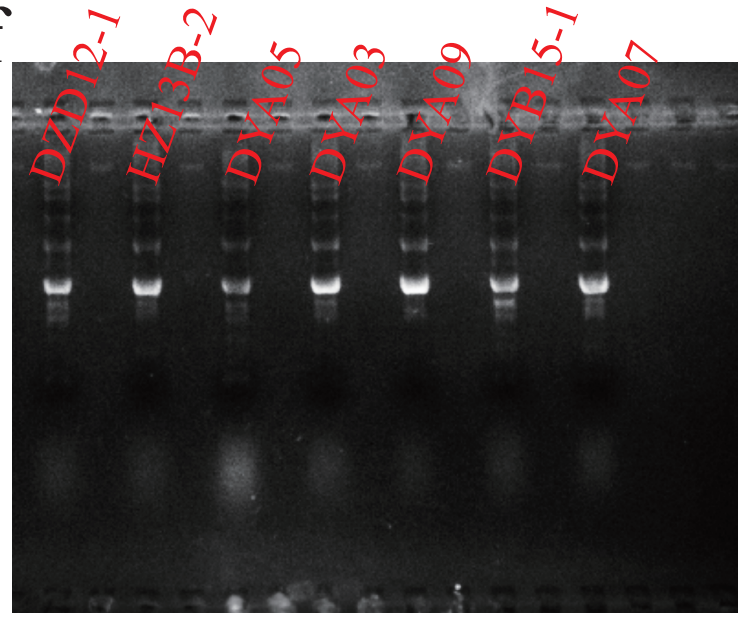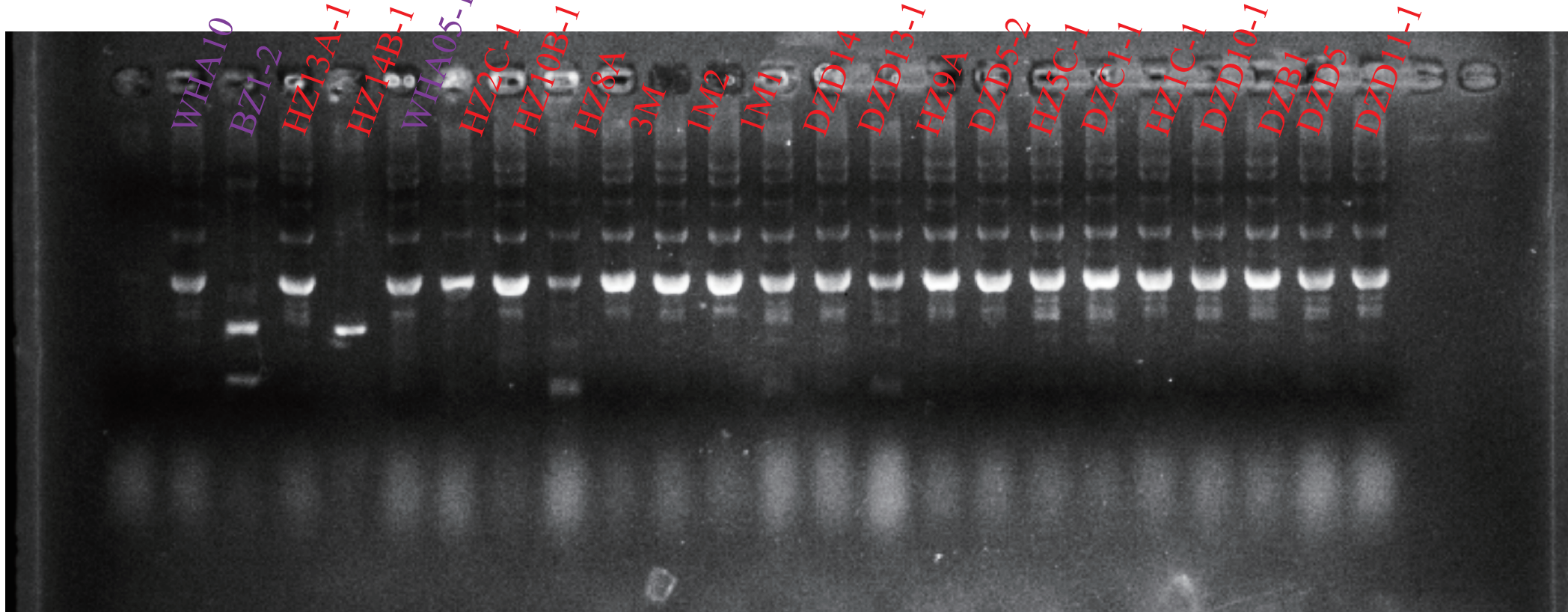

Supplement: Supplementary file 2 [file Data_Sheet_2.PDF]
